# Modulation of DNA Damage Response by Sphingolipid Signaling: An Interplay that Shapes Cell Fate

**DOI:** 10.3390/ijms21124481

**Published:** 2020-06-24

**Authors:** Marina Francis, Alaa Abou Daher, Patrick Azzam, Manal Mroueh, Youssef H. Zeidan

**Affiliations:** 1 Department of Anatomy, Cell Biology and Physiology, Faculty of Medicine, American University of Beirut, Beirut 1107 2020, Lebanon; msf15@mail.aub.edu (M.F.); ara32@mail.aub.edu (A.A.D.); pa27@aub.edu.lb (P.A.); mym13@mail.aub.edu (M.M.); 2 Department of Radiation Oncology, American University of Beirut Medical Center, Beirut 1107 2020, Lebanon

**Keywords:** DNA damage response, double strand breaks, ATM, ionizing radiation, metabolic stress, oxidative stress, p53, sphingolipids, nuclear sphingolipids

## Abstract

Although once considered as structural components of eukaryotic biological membranes, research in the past few decades hints at a major role of bioactive sphingolipids in mediating an array of physiological processes including cell survival, proliferation, inflammation, senescence, and death. A large body of evidence points to a fundamental role for the sphingolipid metabolic pathway in modulating the DNA damage response (DDR). The interplay between these two elements of cell signaling determines cell fate when cells are exposed to metabolic stress or ionizing radiation among other genotoxic agents. In this review, we aim to dissect the mediators of the DDR and how these interact with the different sphingolipid metabolites to mount various cellular responses.

## 1. Introduction

An emerging body of literature indicates that sphingolipids and their metabolizing enzymes are involved in the modulation of the DNA damage response (DDR) [1]. The DNA damage induced by genotoxic stress (ionizing radiation (IR), ultraviolet (UV), chemotherapeutic agents, and metabolic stress) is often coupled to disturbances in the sphingolipids’ homeostasis. Many bioactive sphingolipid metabolites including sphingomyelin (SM), ceramides (Cer), ceramide-1-phosphate (C1P), sphingosine and sphingosine-1-phosphate (S1P) have been proven to regulate important cellular processes such as cell growth, survival, senescence, inflammatory responses, and death [2,3,4]. Therefore, upregulation or downregulation of certain sphingolipids can shift cell fate from survival mode to cell death accordingly. Here, we revise the DDR and the sphingolipid pathway with particular attention paid to the various nuclear sphingolipid metabolites and enzymes. In addition, we shed the light on the interplay between these metabolites and DDR elements to shape cell fate.

## 2. Overview of the DNA Damage Response

Multiple exogenous (IR, UV, and chemotherapeutic agents) or endogenous (oxidative stress, metabolic stress, telomere attrition, and oncogenic mutations) stressors lead to the induction and accumulation of DNA damage [5]. For instance, IR has direct and indirect effects on cellular DNA. The targeted cells lose their ability to further undergo cellular proliferation and eventually die [6]. The ejected charged particles by incident photons can directly hit DNA molecules and disrupt their structure. This direct effect of radiation promotes cell death or induces carcinogenesis if damaged cells survive [7]. IR can also induce reactive oxygen species (ROS) production due to water radiolysis, with water being a major constituent of cells. ROS can attack vital macromolecules including nuclear DNA to induce biochemical damage, also known as indirect effect [8,9]. Most of the IR-induced damage is a consequence of this indirect effect [7]. Given that, the inherent cellular responses to genomic abrasions vary widely depending on the type and extent of inflicted DNA damage.

### 2.1. Types of DNA Damage

DNA damage is mainly constituted of double strands breaks (DSB), single strand breaks (SSB), and base damage (BD).

#### 2.1.1. Double Strand Breaks

DSBs are formed when the chemical bonds of both strands of the DNA molecules are broken. Although the number of induced DSBs is relatively low (around 40 DSBs/Gy of IR), it is the most difficult form of DNA damage to fix. More than 50 min are required to repair 50% of the damage [10]. DSBs are also considered the most lethal forms of DNA lesions. Eventually, unrepaired DSBs result in unrepaired chromosome breaks and micronuclei formation [11]. A single residual DSB can lead to cell death or genomic rearrangements favoring cancer initiation [7,12]. According to Sharma et al., biologically relevant doses of hydrogen peroxide (H_2_O_2_) resulted in a significant increase in oxidative clustered DNA lesions and DSBs formation in DT40 cells at G1 phase [13]. However, some unrepairable DSBs are tolerated by the cell and might not contribute to cell death depending on their localization inside the nucleus and chromatin condensation events [14]. In this review, we mainly elaborate on the repair mechanisms of DSBs.

#### 2.1.2. Single Strand Breaks

SSBs are caused by the cleavage of the phosphodiester bonds in only one strand of the DNA molecule. In fact, 1 Gy dose of IR can induce around 1000 SSBs, of which 50% get repaired within 10 to 20 min following radiation exposure [15,16]. Most ROS-induced DNA lesions are SSBs that can result in erroneous DNA replication or stalling of the replication fork. Some SSBs can eventually lead to DSB formation [17].

#### 2.1.3. Base Damage

BD is a common type of DNA damage where a single dose of 1 Gy can induce around 3000 BDs. These chemical lesions mainly include oxidation, deamination, alkylation as well as hydrolysis that can cause serious genomic aberrations [12,17]. BDs can also be associated with sugar modifications and SSBs [12]. For instance, oxidative stress can induce different types of BDs [18]. ROS may attack any of the four nucleotide bases but most frequently guanine owing to its low oxidation potential. Consequently, the structure of these bases gets disrupted and their pairing properties become altered [19]. These oxidative BDs can cause alterations in gene expression besides mutations that either induce the activation of oncogenes or the inactivation of tumor suppressor genes [20].

### 2.2. Induction of the DNA Damage Response

In response to genomic injuries, cells develop a DNA damage response (DDR) to detect the lesions, indicate their presence, and launch their repair [1,21,22]. The complex signaling pathways involved in the DDR lead to cell cycle arrest and either DNA damage repair or cell death. The DDR serves to maintain the genomic integrity of the cells, which if compromised, would lead to severe disorders [1].

As mentioned earlier, DSBs are classified as the most lethal forms of DNA damage [23]. Since the non-homologous end-joining (NHEJ) pathway is the prevailing repair mechanism of DSBs in quiescent mammalian cells, it is thought to be the predominant element of DDR post-injury as most of the human body cells are in quiescence [24,25]. Furthermore, cells deficient in error prone-NHEJ develop elevated spontaneous chromosomal breaks which become partially repressed after the reduction of cellular oxygen tension [13]. NHEJ repair is based on the direct ligation of both ends of DSBs, mainly during the G1 phase [26]. However, different NHEJ sub-pathways are thought to co-exist, each of which with a distinct repair half-time [27]. On the other hand, homologous recombination (HR) repair pathway uses genetic information from the homologous chromosomes, i.e., from equivalent region found on the second undamaged DNA molecule [26]. This pathway accounts for the systematic repair of only around 15% of IR-induced DSBs [14,27]. Therefore, the lethal effect of IR can be well explained by the accumulation of unrepaired DSBs due to failure in NHEJ [27].

Both endogenous and exogenous stressors are associated with increased risk of SSBs, which are either generated directly or produced as DNA repair intermediates [28]. SSBs must be repaired completely before the initiation of DNA replication to avoid DSBs formation [29]. These SSBs are mainly repaired through base or nucleotide excision repair mechanisms (BER-NER) for a single or multiple and bulky base damage respectively [17]. Oxidative stress may lead to base damage besides SSBs and DSBs. These BDs are mainly repaired through BER pathway that is mediated by DNA *N*-glycosylases [30]. Although the occurrence of SSBs and BD is more frequent than DSBs, their repair mechanisms are more efficient and their contribution to cell lethality is lower.

#### 2.2.1. Detection of Double Strands Breaks (DSBs) and Initiation of the DNA Damage Response (DDR)

The MRN complex, which consists of three subunits, namely Mre11, Nbs1, and Rad50, directly detects DNA damaged sites by binding to the DNA double stranded ends. This complex is responsible for signaling to the DDR upstream kinases including ataxia-telangiectasia mutated protein (ATM) and Rad3-related protein (ATR). As a consequence, ATM and ATR will be recruited to DSBs and RPA-coated SSBs respectively [31,32,33]. In turn, these kinases activate downstream effectors involved in the DDR signaling pathway in order to modulate the progression of the cell cycle and launch the repair [31]. For instance, ATM and/or ATR activate the tumor suppressor protein p53, which is known to be mutated in most cancers, in order to promote cell cycle arrest (p21 activation) or apoptosis (BAX-mediated caspase-3 cleavage) [34]. Moreover, ATM and ATR activate the checkpoint kinases (CHK1 and CHK2) which can either stimulate apoptosis and cell cycle arrest through p53 activation [35] or inhibit Cdc25 to arrest the cell cycle [35]. These checkpoint kinases can further activate BRCA2 to initiate a repair process [36]. Although the activation of ATM by DSBs is a well-established phenomenon, excessive SSBs generation during the repair mechanism can activate ATM independently of DSBs. The activated ATM delays cell cycle progression in a p53/p21-dependent pathway, thus allowing more time for SSBs repair before DNA replication. Consequently, ATM may also contribute to the prevention of DSBs formation [29].

#### 2.2.2. Role of Ataxia-Telangiectasia Mutated Protein (ATM) in DSBs Repair

Members of the phosphatidylinositol-3 kinases family, including ATM, ATR and DNA-dependent protein kinase (DNAPK), are responsible for phosphorylation of the histone variant H2AX near DSBs [37,38]. In effect, γ-H2AX (phosphorylated H2AX) foci formation is a very early step in the DDR of mammalian cells. It plays an essential role in recruiting damage signaling factors to DSBs sites, which are essential for repair induction [37,38]. ATM is a key modulator of the DDR as it directs the activation of cell cycle checkpoints, DNA damage repair and the alteration of cellular metabolism following the induction of DSBs and oxidative stress [39]. Burma et al. demonstrated that ATM was the primary kinase responsible for the rapid phosphorylation of H2AX in response to DNA DSBs and that DNAPK, rather than ATR, took this job in the absence of ATM [40]. However, ATR is the kinase involved in the phosphorylation of H2AX in response to DNA SSBs and replicative stress [41,42]. Therefore, H2AX phosphorylation can be achieved via multiple pathways depending on the DNA damage type.

#### 2.2.3. ATM Nuclear Shuttling

Strong evidence suggests that IR triggers the monomerization of cytoplasmic [14] and nuclear [43] ATM dimers through oxidation reactions resulting in p-ATM (phosphorylated ATM) monomers with increased kinase activity [14,44]. Guo et al. showed that oxidative stress could also trigger the monomerization and activation of ATM irrespective of DSBs induction [45]. Cytoplasmic ATM monomers then can bind to importins through their nuclear localization signals (NLS) and subsequently shuttle into the nucleus [46,47]. This phenomenon is proportionate to the delivered dose [14]. Once inside the nucleus, p-ATM is guided to DSBs sites by signals from the MRN complex. p-ATM phosphorylates the three subunits of the MRN complex as well as the histone variant H2AX resulting in the formation of nuclear foci. These events ensure DSBs recognition and initiation of the repair [47,48,49]. p-ATM nuclear shuttling and foci formation at DSBs is a very rapid process. The maximal number of nuclear p-ATM foci is usually reached within 10 min to 1 h post-injury [50]. However, certain cytoplasmic proteins like mutated huntingtin or tuberous sclerosis complex can bind activated ATM monomers and impede their shuttling towards the nucleus [49,51]. Therefore, DSBs recognition and repair may be influenced by the diffusion of p-ATM monomers as well as ATM re-dimerization or re-association with certain cytoplasmic proteins [14,47,49].

### 2.3. DNA Damage-Induced Cell Death and Senescence

The accumulation of DNA damage provokes multiple responses that lead to cellular senescence or death through various molecular mechanisms. The amount and the type of DNA damage significantly shapes cell fate [52]. With aging, the effectiveness of the DNA repair mechanisms appears to decline, resulting in the accumulation of DNA lesions in tissues [53]. In rodents and humans, altered phenotypes that are common in age-related pathologies, are caused by mutations of some genes involved in the machinery of DNA repair. These alterations include cardiovascular and metabolic disorders such as diabetes, which are linked to increased frequency of malignancies and decreased life expectancy [53,54,55].

Furthermore, cancer therapies involve the induction of unrepairable DNA damage to eradicate cancerous cells. Strong evidence suggests that their therapeutic outcome does not rely on apoptosis alone but rather involves various cell death mechanisms such as mitotic catastrophe, necrosis, and senescence. Cancer cell death does not occur instantaneously, and can take up to weeks from treatment cessation [56]. The guardian of the genome, p53, is a key player in these cellular responses [57]. During tumor progression, pro-apoptotic mechanisms are lost mainly due to impaired functional p53 in more than 50% of human malignant tumors [58,59,60]. The ATM/ATR-activated p53 signals cell cycle arrest and DNA damage repair, thus promoting cell survival. On the other hand, it can also stimulate the elimination of injured cells depending on the cell type and the extent of the DNA damage [61]. The most studied gene targets of p53 are mouse double minute 2 (MDM2), p21, p53 upregulated modulator of apoptosis (PUMA), and Bcl2 associated X (BAX). In normal cells, p53 expression is kept low due to the exhibition of regulatory feedback loops between p53 and MDM2 [62]. MDM2 has E3 ubiquitin ligase activity in addition to nuclear import and export signals. It has many targets including forkhead box O (FOXO) and p53 [63]. Therefore, it tags p53 by ubiquitin at lysine residues in the nucleus causing its nuclear export and subsequent proteasomal degradation [62]. Barak et al. showed that p53 could target MDM2 gene directly [64]; thus, the upregulation of p53 leads to the elevation of MDM2 expression which in turn downregulates p53 [65]. However, tumors may resist apoptosis by retaining functional p53 due to pro-apoptotic genes deactivation (BAX, Apaf1…) or anti-apoptotic genes stimulation (Bcl2, survivin…) [66]. Hence, the regulation of p53 is crucial for determining cell fate post-genotoxic injury.

#### 2.3.1. DNA Damage-Induced Senescence

Senescence is a condition of permanent cell cycle arrest in the G1 phase associated with alterations in gene expression and cellular morphology [52]. Senescent cells are flattened and enlarged cells with a granular cytoplasm that can preserve their metabolic activity and viability [57,67]. The accumulation of senescent stem cells, in particular, leads to impaired homeostasis and tissue regeneration in addition to metabolic dysfunction [68]. These accumulated senescent cells can also lead to p53-independent chronic inflammation in tissues through the release of pro-inflammatory chemokines and cytokines [68]. Moreover, senescence is clinically proven to occur in prostate cancers and desmoids tumors post-radiotherapy as a major p53-dependent mechanism for tumor regression [69,70,71]. Therefore, chronic DDR signaling and senescence hinder damage propagation to the next generation of cells [72].

#### 2.3.2. DNA Damage-Induced Apoptosis

Cellular stress leads to the activation of apoptosis to eradicate aberrant cells when DNA damage repair becomes inefficient [52]. It remains unclear what influences the cell’s decision, whether to launch senescence or apoptosis. Multiple studies demonstrated a cross-talk between senescence and apoptosis mainly at the level of p53 in response to genotoxic stress [73,74]. Activated p53 mounts the transcription of its target genes involved in apoptosis such as PUMA, p53 regulated apoptosis inducing protein 1 (p53AIP1), BAX and apoptotic protease activating factor 1 (Apaf1), to regulate the intrinsic pathway of apoptosis. Moreover, p53 activation can lead to the induction of the extrinsic apoptotic pathway, which is mediated by the upregulation of TRAIL (tumor necrosis factor-related apoptosis-inducing ligand) receptors, death receptors 4 and 5, and CD95 ligand and receptor [75]. Kim et al. identified ROR_α_, an orphan nuclear receptor and a direct p53 target gene, as a stabilizer for p53 and activator of p53 gene transcription. Consequently, ROR_α_ resulted in the activation of the p53 downstream effectors involved in apoptosis [76]. Caspase-2 also plays a role in linking DNA damage to the pathways of apoptosis. The upregulation of PIDD (p53-induced protein with a death domain) leads to the spontaneous cleavage and activation of caspase-2 in the nucleus [77] and consequently sensitizes cells to genotoxins-induced apoptosis [78]. For instance, there is an association between radiation-induced apoptosis and the activation of ATM/p53/BAX/cytochrome c/caspases pathway [79]. However, DNA damage-induced apoptosis might be p53-dependent or independent based on the damage and cell types [52].

#### 2.3.3. DNA Damage-Induced Mitotic Catastrophe

Mitotic catastrophe designates cell death occurrence during or as a consequence of abnormal mitosis [80]. This results in aberrant segregation of chromosomes, followed by the generation of huge cells that hold abnormal nuclear morphologies, multiple nuclei [81,82,83,84], and/or multiple micronuclei [85]. It has been suggested that mitotic catastrophe occurs as a result of defective cell cycle checkpoints or aggregated DNA damage that triggers p53 mutation or inactivation [57,86]. p53-dependent centrosomes proliferation can also cause mitotic catastrophe after DNA damage induction and defective repair [87,88,89,90,91]. Delayed cell death induction by radiotherapy in solid tumors is mainly caused by the mitotic catastrophe [57] associated with p53/cytochrome c/caspases pathway [92]. Thus, mitotic catastrophe is considered an important cell death mechanism inflicted by DNA damage.

#### 2.3.4. DNA Damage-Induced Necrosis

In contrast to apoptosis, necrosis is an uncontrolled cell death mechanism. Necrosis is characterized by various morphological changes, including increased cell volume, swelling of organelles, ruptured plasma membranes, and loss of intracellular components. It can be mediated by several catabolic and signal transduction pathways including TNFα/PARP/JNK/caspases [93,94]. Necrosis is a form of death that involves immunological activation and pro-inflammatory responses through the release of damage-associated molecular patterns like high mobility group (box1) and heat shock proteins (HSPs) [95,96]. As mitotic catastrophe is usually followed by necrosis, local inflammation tends to develop post-radiotherapy [97]. Given that, necrosis is one of the major contributors to cell death in response to genotoxic injuries.

## 3. Overview of Sphingolipids

Sphingolipids belong to a class of lipids characterized by a sphingosine backbone, which is an amino-alcohol compound of 18 carbon atoms. The sphingosine backbone is synthesized from non-sphingolipid precursors in the endoplasmic reticulum (ER). The diversity of sphingolipids arises from distinct variations in this basic structure [98].

Sphingolipids are no longer thought to be only major structural components of biological membranes but have recently proven to be, along with their active metabolites (sphingomyelin, ceramide, sphingosine, sphingosine-1-phosphate, ceramide-1-phosphate), key players in various human diseases [99,100]. These bioactive signaling molecules mediate various important biological processes such as cell growth, survival, senescence, inflammatory responses, and death by regulating their downstream targets [4,101]. For instance, Golgi-associated retrograde protein complex (GARP) is a mediator of retrograde vesicular transport from the endosome to Golgi, and thus important for the regulation of sphingolipids’ recycling between the plasma membrane and endosomes. GARP deficiency ultimately leads to the development of progressive cerebello-cerebral atrophy type 2 (PCCA2) [102]. This severe neurodegenerative disease is attributed to dysfunctional lysosomes and dysregulated sphingolipid metabolism [102]. Niemann-Pick disease type C (NPC) is a lysosomal storage disease caused by mutations in lysosomal proteins NPC1 or 2. It involves the accumulation of sphingosine, sphingomyelin, glycosphingolipids, and cholesterol [103,104]. In Niemann–Pick disease, the accumulation of sphingomyelin is caused by acid sphingomyelinase deficiency [105]. Sphingosine contributes to lysosomal calcium release through two-pore channel 1 (TPC1) in normal fibroblasts. In contrast, cells derived from NPC patients exhibit reduced lysosomal calcium release due to accumulated sphingosine [104]. Moreover, sphingolipids are associated with various genetic (Gaucher disease) and non-genetic (diabetic nephropathy and focal segmental glomerulosclerosis) glomerular diseases. The dysregulation of sphingolipids in podocytes disrupts their proper functioning and subsequently compromises the glomerular filtration barrier [106]. Given that, sphingolipids altered metabolism mediates various inherited and non-inherited human diseases.

### 3.1. Sphingolipids Metabolic Pathway

**Figure 1:**
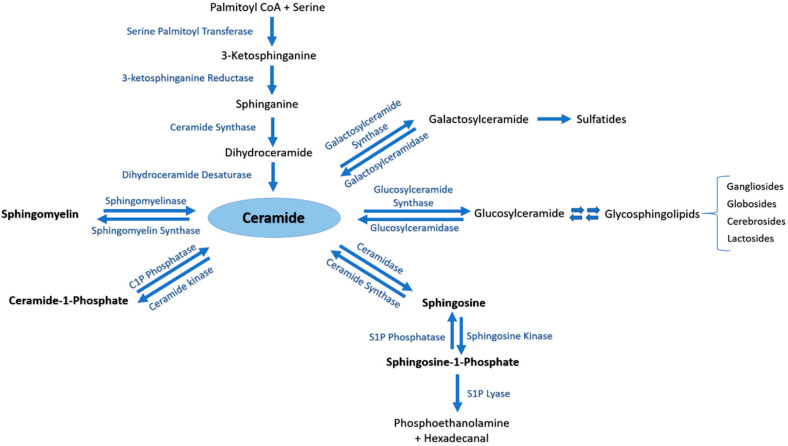
The sphingolipid metabolic pathway. Ceramide is the central metabolite generated in the sphingolipid metabolism by three distinct pathways. Ceramide de novo synthesis consists of Palmitoyl-CoA condensation with serine by the action of serine palmitoyl transferase, followed by a set of reduction and acetylation reactions to generate ceramide. Sphingomyelin (SM) catabolism generates ceramide through the action of sphingomyelinases. The salvage pathway involves N-acylation of fatty acids with a sphingosine backbone to produce ceramide by ceramide synthases. Ceramide can be further phosphorylated to ceramide-1-phosphate (C1P) by ceramide kinase, or converted into complex glycosphingolipids by glucosylceramide or galactosylceramide synthases. Ceramidase is responsible of catabolizing ceramide into sphingosine, which may be further metabolized by sphingosine kinases to generate sphingosine-1-phosphate (S1P).

Ceramide (Cer) is the central metabolite generated within the sphingolipid metabolism through three different pathways. (i) Ceramide de novo synthesis: Palmitoyl-CoA is condensed with serine by the action of serine palmitoyl transferase, followed by a set of reduction and acetylation reactions to generate ceramide; (ii) Sphingomyelin (SM) catabolism: SM is catabolized by sphingomyelinases to generate ceramide; (iii) Salvage pathway: *N*-acylation of fatty acids with a sphingosine backbone produces ceramide through the action of ceramide synthases [1]. The generated pro-apoptotic Cer can be phosphorylated by ceramide kinase into ceramide-1-phosphate (C1P) in trans-Golgi or plasma membranes. C1P plays an important role in inflammatory responses, cell survival and proliferation [98,99]. Afterwards, C1P can be dephosphorylated by C1P phosphatases or other unspecific lipid phosphate phosphatases (LPP family) [98,107]. Cer is also utilized to generate two major groups of complex glycosphingolipids. Glucosylceramide synthase generates glucosphingolipids by adding glucose as the first residue to Cer at C1 hydroxyl position, whereas galactosylceramide synthase generates galactosphingolipids by adding galactose to Cer [98]. Moreover, Cer can be catabolized by ceramidases into sphingosine which promotes cell cycle arrest and apoptosis. In its turn, sphingosine can be phosphorylated by sphingosine kinases into the pro-survival lipid sphingosine-1-phosphate (S1P) [101]. S1P can be dephosphorylated by S1P phosphatases [108,109] or unspecific LPP [110]. The generated sphingosine can be further used to produce Cer or S1P [109]. S1P lyase (SPL) is considered as the last enzyme in the sphingolipid catabolic pathway because it can irreversibly break down S1P into phosphoethanolamine and hexadecenal [111] (Figure 1).

### 3.2. Sphingolipids in the Nucleus

It took a long time to dismiss the hypothesis that the lipids found in the nuclear fraction result from contamination during extraction procedures. However, several years of dedicated research confirm that lipids are minor components of the nucleus (around 5% by weight) [112,113]. Under various physiological and pathological conditions, the composition, metabolism and behavior of these nuclear lipids are independent of the other lipids in cellular membranes and organelles [112,114]. It is of note that some exogenous stimuli influence only intranuclear signaling [115,116] while others can influence both the nuclear and cytoplasmic signaling [117,118]. In the nuclear fraction, lipids could be either polar or non-polar and consist of glycerophospholipids, plasmalogens, sphingolipids, gangliosides, cholesterol, arachidonic acid, and eicosanoids [112]. Their active metabolism is maintained by nuclear lipid enzymes which take responsibility of their anabolic and catabolic reactions [114]. Initially, the function of nuclear lipids was thought to be restricted to structural support maintenance of the nuclear membranes (nuclear envelope (NE)) as they contain the bulk of lipids [119]. However, besides the nuclear membranes, bioactive lipids were also identified in other subnuclear domains including the nuclear matrix [120], chromatin [121,122], and nucleolus [123]. Structurally, the NE consists of outer and inner nuclear membranes of distinct lipidomic profiles. The outer membrane is continuous with the ER, whereas the inner membrane is associated with the nuclear lamina and chromatin [124]. These nuclear membranes are separated by a perinuclear space and they are perforated by the nuclear pore complexes (NPC). The latter control nucleo-cytoplasmic communications mainly by regulating the bidirectional shuttling of ions, nucleotides, RNA and proteins [125]. Albi et al. demonstrated that the nuclear membranes’ permeability and fluidity are heavily dependent on their lipid composition, in particular phosphatidylcholine (PC), sphingomyelin (SM) and cholesterol (CHO) [126]. Moreover, the inner membrane expresses a GM1 ganglioside-linked Na^+^-Ca^2+^ exchanger that is responsible of mediating the transfer of nuclear Ca^2+^ to the perinuclear space as a cytoprotective mechanism [127]. Gangliosides are complex glycosphingolipids, which are mostly abundant in the central nervous system and involved in the regulation of nuclear calcium homeostasis [113]. The lipid content of the nuclear matrix is essential for conveying and maintaining its rigidity. It constitutes an anchor that organizes the chromatin and controls various important endonuclear events [128]. In fact, nuclear lipids are involved in multiple processes like DNA replication, transcription, splicing, and repair as well as Ca^2+^ homeostasis [129]. Therefore, stress-induced alterations in lipid metabolism can modulate cell growth, survival, differentiation, senescence and death [114].

Many studies have identified sphingolipids as important modulators of key nuclear processes. So far, various subnuclear compartments including the NE, nuclear matrix, nucleolus and chromatin have been described to host various sphingolipid species [113,123,130,131,132,133,134,135,136]. Although nuclear pores should permit nucleo-cytoplasmic exchange of sphingolipids, many of these nuclear metabolites are in dynamic state and undergo turnover. Therefore, the nuclear localization of sphingolipid metabolizing enzymes has been demonstrated. To this end, the utilization of several analytical, biochemical and microscopic techniques led to the identification and quantification of various nuclear sphingolipid species along with their metabolizing enzymes [137]. In fact, sphingomyelin (SM) is the dominant nuclear sphingolipid variant [119]. Through its metabolism, SM gives rise to ceramides, sphingosine, and S1P, which in turn give rise to other metabolites. Within the scope of this review, we describe and summarize the diverse functions of these nuclear sphingolipids based on their localization (Table 1).

#### 3.2.1. Nuclear Sphingomyelin and Metabolizing Enzymes

SM is the most abundant nuclear sphingolipid. It is primarily found in the nuclear envelope and to a lesser extent in the nuclear matrix and chromatin [113]. Besides SM, the nuclear membrane contains phosphatidylcholine (PC) and cholesterol (CHO). These are the important lipids that regulate the structure, function, and fluidity of nuclear membranes [138]. SM and CHO increase a membrane’s rigidity, whereas PC increases its fluidity [125]. Therefore, a high SM-CHO/PC ratio will decrease the fluidity of the nuclear membrane and vice versa. As the fluidity of the nuclear membrane increases, the size of the nuclear pores changes allowing increased nucleo-cytoplasmic transport such as that of mRNA during cell proliferation [138]. Moreover, nuclear SM can either stabilize and/or destabilize the DNA molecules by influencing the helical to non-helical transition and vice versa [139]. The SM-DNA interaction is plausible due to the zwitterionic nature of SM. Its positively charged trimethylammonio group can bind to DNA anionic groups, whereas its negatively charged phosphate group repels the negatively charged phosphate of the DNA [112]. At low levels, the non-polar fatty acids of SM bind to the internal hydrophobic centers of helical DNA providing their stabilization. Conversely, increased SM concentration leads to space competition for the non-polar fatty acids. This change alters their binding to DNA and results in DNA molecule opening followed by rapid denaturation [112]. It has been proposed that SM also binds to and stabilizes double-stranded RNA in the nucleus and prevents its digestion by RNases [140,141]. In addition, SM is an essential component for the maintenance of the nuclear structure since it preferentially localizes within the peri-chromatin region. This is supported by the observation that sphingomyelinase microinjections into living cells’ nuclei resulted in fast corrosion of the intranuclear architecture [142,143].

The metabolism of nuclear SM is independent from the Golgi complex and ER. Multiple factors such as stress, high fat diet, cell cycle and tissue regeneration can alter the levels of SM in the nucleus [124]. The enzymes responsible for SM synthesis and breakdown have been detected in nuclear fractions [124]. *De-novo* nuclear SM synthesis requires the production of Cer, followed by its conversion to SM by the action of SM synthase 1 or 2. The latter step requires phosphocholine derived from nuclear PC which is found in the NE or chromatin [144,145]. On the other hand, neutral sphingomyelinase, available in the nuclear envelope [130], nuclear matrix [120], and chromatin [146] of rat liver nuclei, metabolizes SM into pro-apoptotic ceramides. Reverse SM synthase was also detected in rat liver chromatin, which catalyzes the transfer of phosphocholine from SM into DAG, a mitogenic second messenger, forming PC [144]. Therefore, SM synthase and sphingomyelinase can modulate cell proliferation or death by regulating Cer to DAG ratio of chromatin.

#### 3.2.2. Nuclear Ceramide, Ceramide-1-Phosphate and Metabolizing Enzymes

Cer is the central metabolite generated within the sphingolipid pathway. It serves as a precursor for complex sphingolipids production (SM and glycosphingolipids) and in turn can be metabolized to other bioactive species (sphingosine, C1P or S1P) [129]. After overexpression in HEK-293 cells, Cer synthases could be highly detected in the ER and NE [147,148,149,150]. Nuclear ceramidase activity was also reported in liver nuclear membranes, thus allowing further Cer metabolism [151]. Several studies showed that nuclear ceramides are key mediators of cell cycle arrest and apoptosis. Multiple exogenous stressors can alter the nuclear levels of Cer such as serum starvation, high-fat diet, bacterial infections, and apoptosis-inducing mediators (e.g., Fas ligand) [124,152]. For instance, Albi and colleagues reported that serum starvation was associated with nuclear Cer upregulation during the early phase of apoptosis. This was followed by extranuclear sphingomyelinases activation and cytoplasmic Cer accumulation during the late phase of apoptosis [153]. A high fat diet also resulted in increased nuclear ceramide levels by three-fold in rat liver nuclei along with the elevation of saturated fatty acid species (C:14, C:16, C:18) [154]. It remains unclear whether Cer nucleo-cytoplasmic shuttling is feasible via binding to Cer transport protein CERT and FAPP2 [155,156].

Cer can be phosphorylated into C1P by the action of ceramide kinase (CERK) previously reported in ER/Golgi organelles [157]. Then, C1P transfer protein (CPTP) transports C1P to the cytoplasmic membrane and other subcellular organelles including the nucleus [158]. Prior work detected nuclear import and export signals in the protein sequence of CERK [159]. It is plausible that nuclear ceramides may be further converted into C1P, however that remains to be fully established.

#### 3.2.3. Nuclear Sphingosine, Sphingosine-1-Phosphate and Metabolizing Enzymes

Sphingosine levels, whether in whole cells or nuclear extracts, are much lower than Cer [133]. Nuclear ceramidases allow the hydrolysis of Cer into sphingosine which in turn can be converted into Cer by the action of Cer synthases [129,133]. Nuclear sphingosine is an important regulator of gene transcription. Sphingosine modulates the transcription of CYP17 and it is considered as a regulatory ligand for steroidogenic factor (SF-1) [160]. Under basal conditions, nuclear sphingosine binds to SF-1 with several co-repressors including Sin3A and histone deacetylase (HDAC). The stimulatory signals of the adrenocorticotropin hormone (ACTH) release sphingosine from bounded SF-1 through the activation of protein kinase A. Subsequently, the transcription of genes implicated in steroid hormone synthesis from cholesterol precursor will be initiated [161,162].

In addition, sphingosine levels can be modulated by the action of sphingosine kinases (SK) which phosphorylate sphingosine to sphingosine-1-phosphate (S1P). There are two isoforms of sphingosine kinases, SK1 and SK2 which differ by their subcellular localizations and functions. SK1 is mainly located in the cytoplasm due to its two functional nuclear export signals and regulates cell proliferation and growth. Conversely, SK2 is mainly located in the nucleus, due to the nuclear localizing signal at its N-terminus, and modulates apoptosis [163,164]. Both sphingosine kinases get altered after stimulation by growth and survival factors. They become subjected to post-translational modifications, translocations, protein-protein and lipid-protein interactions resulting in increased intracellular S1P levels [165]. Primally, nuclear SK activity was detected in the NE and nucleoplasm of Swiss 3T3 cells. This kinase activity got upregulated by the platelet derived growth factor and promoted cell cycle progression toward the S phase [118]. Therefore, S1P might be implicated in the regulation of cell cycle. In MCF-7 breast cancer cells, SK2 interacts with the histone variant H3 in chromatin and induces its acetylation. Thus, intranuclear S1P can exert epigenetic modulations of gene transcription. The nuclear S1P and dihydro-S1P can bind to the active sites of HDAC1 and 2 and consequently inhibit their activities [136]. Moreover, SK2 associates with HDAC at the promoter regions of p21 and c-*fos* genes resulting in histone acetylation, which favors their gene transcription and subsequent cell cycle arrest and apoptosis [136]. Recently, Selvam et al. suggested that S1P can bind and stabilize the human telomerase [166]. S1P can act as an intracellular messenger or an extracellular ligand for a family of five isoforms of G protein-coupled receptors (S1PR1-5) [167]. IHC and ICC techniques allowed the detection of all five isoforms of S1PR in both the cytoplasm and nucleus of healthy and cancerous human tissues of several organs [168]. All together, these studies suggest that S1P is a master regulator of cell proliferation and anti/pro-apoptotic processes.

To terminate S1P signaling at the ER, the cells opt either sphingosine phosphatases (SPP1-2) which dephosphorylate S1P into sphingosine [169] or S1P lyase (SPL) which terminally hydrolyzes S1P into ethanolamine phosphate and hexadecenal [111]. Schwiebs et al. reported that SPP-1 is expressed in the nuclear compartment of naïve dendritic cells and gets translocated to the cytoplasm upon inflammation [170]. Recently, Ebenezer et al. confirmed the nuclear localization of SPL in lung epithelial cells [171], as well as the crucial role of the generated nuclear hexadecenal in histone acetylation through interaction with HDAC1-2 [172]. The exact catabolic mechanisms of nuclear S1P remain to be fully elucidated in various tissues and cell lines.

## 4. Role of Sphingolipids in the DNA Damage Response

**Figure 2:**
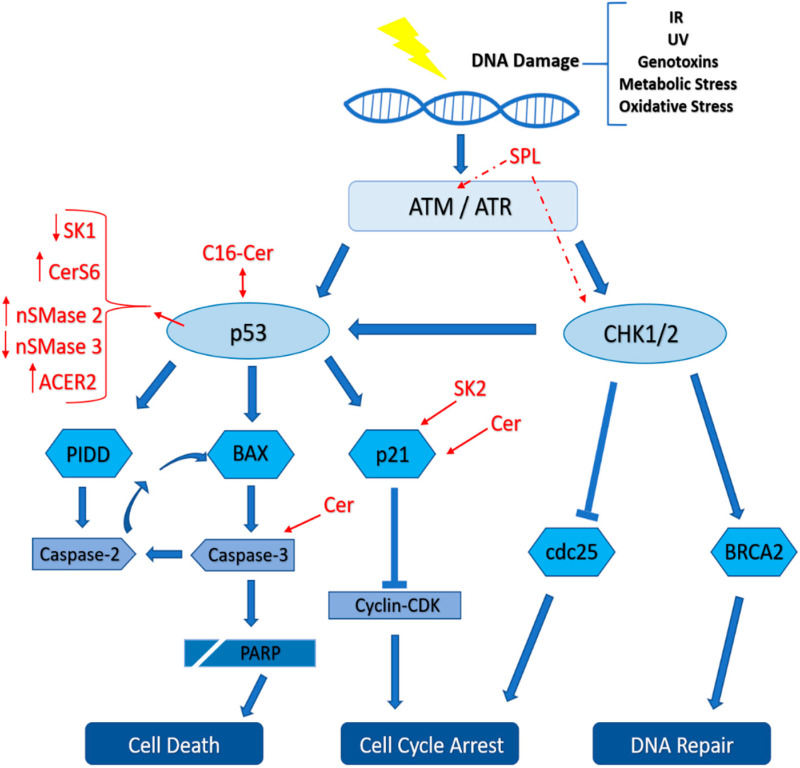
Interplay between DNA damage response (DDR) and sphingolipids to shape cell fate after DNA damage. After genotoxic injury, the DDR develops to sense the damage and amplify the transmitted signaling cascade. The transiently activated cell cycle arrest allows DNA repair. However, persisting unrepaired damage triggers cellular senescence or death to hinder damage propagation to the next generation of cells. The sphingolipid metabolism interacts mainly with p53 along with other elements of the DDR to determine the injured cell fate. ACER2: alkaline ceramidase 2, Cer: ceramide, CerS: ceramide synthase, nSMase: neutral sphingomyelinase, SK: sphingosine kinase, SPL: S1P lyase, double-headed arrow: interaction and activation, full arrow: upregulation and activation, and dashed arrow: downregulation.

After reviewing the DDR and the various nuclear sphingolipid metabolites, we discuss how these two entities interplay in order to determine cell fate post-injury (Figure 2). Various chemotherapeutic drugs and DNA damaging agents target sphingolipid metabolizing enzymes. Strong evidence suggests that lipids are involved in DDR and determining cell fate [1]. Most of cancer treatments lead to Cer generation which is implicated in cell death response [173]. However, cancer cells tend to develop survival strategies like generating the pro-survival sphingolipid metabolite S1P after the phosphorylation of sphingosine generated by Cer hydrolysis [174]. Hence, the regulation of these metabolites production is of significant importance in determining the cells’ fate in response to DNA damage [1].

Dbaibo et al. showed that p53 is involved in Cer-induced apoptosis. The accumulation of Cer in Molt4 lymphocyte leukemia cells post-irradiation and actinomycin D was p53-dependent since increased p53 preceded Cer up-regulation. Indeed, Cer accumulation can be impeded by p53 inhibition [175]. p53 is a downstream effector activated by ATM in DDR and numerous indications implicate its involvement in Cer accumulation. Prior work demonstrated that AT cells with a mutated ATM gene are resistant to IR-induced apoptosis. These cells maintained the first phase of Cer accumulation by acid sphingomyelinase but lost the second peak mediated by Cer synthase [176]. Thus, ATM mediates Cer synthase activation but not that of acid sphingomyelinase. Further studies point at the involvement of neutral sphingomyelinases 2 and 3 in DDR. ATM and p53 activate neutral sphingomyelinase 2 and down-regulate neutral sphingomyelinase 3 in order to induce apoptosis [177,178,179]. Furthermore, it has been shown that IR induces caspase-3 and PARP cleavage through Cer. Caspase-3 inhibition doesn’t affect the levels of Cer whereas Cer depletion prevents the cleavage of caspase-3 and PARP [176,180]. These results suggest that Cer up-regulation is upstream of caspase-3 cleavage. Ceramides have been also implicated in cell cycle arrest during DDR. For instance, accumulation of Cer can arrest the cell cycle either at G0/G1 phase mediated by retinoblastoma protein (Rb) [181] or at G2 phase through the activation of p21 [182]. Others reported that human alkaline ceramidase 2 (ACER2) is a novel direct target gene of p53 that mediates the DDR [183]. Upregulation of ACER2 reduced the levels of Cer while accumulating sphingosine and S1P in H1299 cells. Extensive IR-induced DNA damage hyperactivated the p53-ACER2 pathway in HCT116 cells. This hyperactivation led to cell death because of a high pro-apoptotic sphingosine to pro-survival S1P ratio. In contrast, low levels of DNA damage moderately activated the p53-ACER2 pathway favoring cell cycle arrest and senescence. The pro-apoptotic and pro-senescence signals of sphingosine and Cer were balanced with the pro-survival and pro-proliferative signals of S1P [183]. In cancer cells, folate stress leads to the upregulation of C16-Cer coupled to a transient increase in Cer synthase 6 in a p53/PUMA-dependent manner [184]. Recent work proved a direct and highly specific interaction between p53 and C16-Cer. Under metabolic stress induced by serum or folate starvation, this interaction displaces MDM2 from p53 leading to the accumulation and activation of p53 [185]. However, the relationship between p53 and Cer remains elusive as many studies supported functional roles for Cer both upstream and downstream of p53.

The levels of the pro-survival bioactive lipid S1P are also involved in DDR and the determination of cell fate [1]. A link between p53 and SK1, which catalyzes S1P production, was established after treating Molt-4 leukemia cells with multiple chemotherapeutic agents and γ-rays. The DDR leads to a decrease in the protein but not mRNA levels of SK1 coupled to p53 up-regulation [186]. Conversely, SK2 was shown to exacerbate the apoptotic response and its overexpression was associated with the induction of apoptosis. In addition, SK2 was proven as critical for p21 expression which was needed for cell cycle arrest in a p53-independent route [164,187]. These results could be potentially explained by the fact that mainly SK1 is cytoplasmic and SK2 is nuclear. Hence, increased SK2 expression and intranuclear production of S1P can inactivate HDACs and promote the transcription of genes involved in cell cycle arrest and apoptosis. On the other hand, SPP1 knockdown had a protective role against DNA damage and cell death induced by daunorubicin in MCF7 cells [188]. Furthermore, augmenting SPL activity through its overexpression or by treatment with etoposide favored apoptosis through caspase-3, annexin-V, PARP and nuclear condensation [189]. Similarly, SPL modulates DNA repair, G2 cell cycle arrest, and apoptosis post-irradiation. SPL upregulation exacerbates stress-induced accumulation of Cer through acid sphingomyelinase. Interestingly, overexpression of SPL results in decreased ATM and checkpoint kinase 1 protein levels when compared to control cells [190]. Taken together, these findings reveal the importance of sphingolipids in mediating the DNA damage response.

Our group previously showed that the specific expression of sphingomyelin phosphodiesterase acid-like 3b (SMPDL3b) in podocytes to be a modulator of radiation stress signaling [191]. Strong evidence suggests that SMPDL3b is critical for preserving podocytes proper functioning [192]. Novel work pinpointed that SMPDL3b regulates C1P to Cer levels in podocytes by interacting with CERK and C1P [193,194]. Furthermore, IR results in a time-dependent drop in the protein levels of SMPDL3b, downregulation of sphingosine and S1P, upregulation of various pro-apoptotic Cer species, and loss of podocytes’ filopodia [191]. Conversely, SMPDL3b overexpression confers radioresistance by partially reversing these changes and enhancing DNA damage repair evidenced by reduced γ-H2AX nuclear foci in comparison to wild-type podocytes post IR [191]. It remains of great interest to elucidate the proper mechanism by which SMPDL3b modulates the DDR.

In a nutshell, all non-surgical cancer therapies aim to eradicate tumor cells while sparing normal tissues through complex cell signaling pathways. Research over the past few decades has confirmed that the stress induced by these therapies involves the accumulation of ceramide. However, any dysregulation in this process, due to either decreased generation or increased metabolism of ceramide, confers resistance against these therapies [195]. From this perspective, emerging therapeutic and clinical interventions are under investigation to maximize the positive outcomes of these therapies by a combinatorial approach. For instance, as recombinant human acid sphingomyelinase (rhASM) was previously evaluated in patients with Niemann–Pick disease, the idea of its administration in cancer therapies flourishes. rhASM might be used to induce pro-apoptotic ceramide levels beyond the tolerance of cells. This treatment is more likely to affect tumors than normal tissues [196]. Moreover, a recent study reported that gentamicin, a commonly used anti-microbial drug, can potentially play a role in cancer therapies. The administration of gentamicin highly upregulated acid sphingomyelinase and induced apoptosis in human gastric cancer cells [197]. As cancer cells can develop survival strategies like generating the pro-survival S1P, inhibitors for both SK1 and SK2 were developed. However, sphingosine kinase inhibitors exhibited some downstream off-target effects such as inhibiting ERK and Akt pathways [198]. Hence, further studies should address the development of more specific sphingosine kinase targets for possible clinical trials. Interestingly, the total plasma levels of ceramide, measured in early days after the combined treatment of radio-chemotherapy, can predict tumor responses in patients with liver and lung metastases of colorectal cancer. It allows the identification of patients with high risks of metastases [199]. Therefore, successful discoveries of sphingolipid therapeutic targets or sphingolipid biomarkers of tumor response will potentially enhance the outcomes of standard of care therapies.

## 5. Conclusions

Up to now, a large body of evidence supports the interplay between the DDR and the sphingolipid metabolic pathway in determining cell fate after exposure to genotoxins or metabolic stress. These findings elicit sphingolipids as master modulators of the overall mechanism by which cells respond to genotoxic injuries. Further studies are needed to clarify some debatable theories and to further understand the complex interactions between the various sphingolipid metabolites and the DDR mediators. For instance, although SK2 was reported to mediate cell cycle arrest and apoptosis [164,187], downregulation of SK2 but not SK1 was effective in the suppression of tumor cell proliferation and migration [200]. Despite the numerous studies that emphasized important roles of sphingolipids in cancer and metabolic diseases, a very limited number of sphingolipid-targeting drugs entered clinical trials. Complete understanding of these bioactive metabolites and enzymes remains to be fully elucidated. The first-in-class clinical inhibitor of SK2, ABC29464, successfully achieved phase I clinical trial and proceeded to phase II as an anti-cancer drug [201]. Therefore, new effective sphingolipid therapeutic targets are expected to develop as a result of the numerous emerging studies in the field. These potential discoveries are predicted to advance the standard of care therapies by overcoming tumor resistance and developing new effective diagnostic and prognostic sphingolipidomic-based tests.

## Figures and Tables

**Table 1:** Nuclear sphingolipid metabolites and metabolizing enzymes. This table recapitulates the various nuclear sphingolipid metabolites and enzymes detected in the nuclear compartment with a brief description of their important nuclear functions. NE: nuclear envelop, dsRNA: double stranded RNA.

Nuclear Sphingolipid Metabolites	Nuclear Sphingolipid Producing Enzymes	Nuclear Sphingolipid Degrading or Converting Enzymes	Main Nuclear Functions
*Sphingomyelin*	Sphingomyelin synthase	Reverse sphingomyelin synthase Neutral sphingomyelinase	Maintenance of NE and nucleoplasm structure Regulation of NE permeability and Fluidity Stabilization of DNA and dsRNA
*Ceramide*	Ceramide synthase Ceramide desaturase Neutral sphingomyelinase	Ceramidase Ceramide kinase	Regulation of Cell cycle arrest, Senescence, and Apoptosis
*Ceramide-1-phosphate*	Ceramide kinase	C1P phosphatase	Regulation of cell growth and survival
*Sphingosine*	Ceramidase	Ceramide synthase Sphingosine kinase 2	Regulation of gene transcription and apoptosis
*Sphingosine-1-phosphate*	Sphingosine kinase 2	S1P lyase S1P phosphatase	Epigenetic modulation of gene transcription Regulation of cell cycle progression and apoptosis Stabilization of human telomerase

## Abbreviations

Apaf1	Apoptotic protease activating factor 1
ATM	Ataxia telangiectasia mutated
ATR	Ataxia telangiectasia and Rad-3 related
BD	Base damage
Cer	Ceramide
CERK	Ceramide kinase
CHO	Cholesterol
C1P	Ceramide-1-phosphate
DDR	DNA damage response
DNAPK	DNA-dependent protein kinase
DSB	Double strand break
Gy	Gray (absorbed dose)
HR	Homologous recombination
IR	Ionizing radiation
MDM2	Mouse double minute 2
NHEJ	Non-homologous end joining
PC	Phosphatidylcholine
PUMA	p53 upregulated modulator of apoptosis
p53AIP1	p53 regulated apoptosis inducing protein 1
ROS	Reactive oxygen species
SK	Sphingosine kinase
SM	Sphingomyelin
SMPDL3b	Sphingomyelin phosphodiesterase acid like-3b
S1P	Sphingosine-1-phosphate
SPP	S1P phosphatase
SSB	Single strand break
TRAIL	TNF-related apoptosis-inducing ligand

## References

[1] Carroll B., Donaldson J.C., Obeid L. (2015). Sphingolipids in the DNA damage response. Adv. Biol. Regul..

[2] Presa N., Gomez-Larrauri A., Dominguez-Herrera A., Trueba M., Gomez-Muñoz A. (2020). Novel signaling aspects of ceramide 1-phosphate. Biochim. Biophys. Acta (BBA) Mol. Cell Biol. Lipids.

[3] Taniguchi M., Okazaki T. (2014). The role of sphingomyelin and sphingomyelin synthases in cell death, proliferation and migration—From cell and animal models to human disorders. Biochim. Biophys. Acta (BBA) Mol. Cell Biol. Lipids.

[4] Hannun Y.A., Obeid L.M. (2018). Sphingolipids and their metabolism in physiology and disease. Nat. Rev. Mol. Cell Biol..

[5] López-Otín C., Blasco M.A., Partridge L., Serrano M., Kroemer G. (2013). The hallmarks of aging. Cell.

[6] Jackson S.P., Bartek J. (2009). The DNA-damage response in human biology and disease. Nature.

[7] Saha G.B. (2012). Physics and Radiobiology of Nuclear Medicine.

[8] Hall E.J., Giaccia A.J. (2006). Radiobiology for the Radiologist.

[9] Azzam E.I., Jay-Gerin J.-P., Pain D. (2012). Ionizing radiation-induced metabolic oxidative stress and prolonged cell injury. Cancer Lett..

[10] Negritto C. (2010). Repairing double-strand DNA breaks. Nat. Educ..

[11] Cornforth M.N., Bedford J.S. (1987). A quantitative comparison of potentially lethal damage repair and the rejoining of interphase chromosome breaks in low passage normal human fibroblasts. Radiat. Res..

[12] Bauer N.C., Corbett A.H., Doetsch P.W. (2015). The current state of eukaryotic DNA base damage and repair. Nucleic Acids Res..

[13] Sharma V., Collins L.B., Chen T.-h., Herr N., Takeda S., Sun W., Swenberg J.A., Nakamura J. (2016). Oxidative stress at low levels can induce clustered DNA lesions leading to nhej mediated mutations. Oncotarget.

[14] Bodgi L., Foray N. (2016). The nucleo-shuttling of the atm protein as a basis for a novel theory of radiation response: Resolution of the linear-quadratic model. Int. J. Radiat. Biol..

[15] Isaksson M., Raaf C.L. (2017). Environmental Radioactivity and Emergency Preparedness.

[16] Thompson L.H. (2012). Recognition, signaling, and repair of DNA double-strand breaks produced by ionizing radiation in mammalian cells: The molecular choreography. Mutat. Res./Rev. Mutat. Res..

[17] Lindahl T. (1993). Instability and decay of the primary structure of DNA. Nature.

[18] Cadet J., Wagner J.R. (2013). DNA base damage by reactive oxygen species, oxidizing agents, and uv radiation. Cold Spring Harb. Perspect. Biol..

[19] Bokhari B., Sharma S. (2019). Stress marks on the genome: Use or lose?. Int. J. Mol. Sci..

[20] Klaunig J.E., Kamendulis L.M., Hocevar B.A. (2010). Oxidative stress and oxidative damage in carcinogenesis. Toxicol. Pathol..

[21] Harper J.W., Elledge S.J. (2007). The DNA damage response: Ten years after. Mol. Cell.

[22] Rouse J., Jackson S.P. (2002). Interfaces between the detection, signaling, and repair of DNA damage. Science.

[23] Jeggo P., Löbrich M. (2007). DNA double-strand breaks: Their cellular and clinical impact?. Oncogene.

[24] Woodbine L., Gennery A.R., Jeggo P.A. (2014). The clinical impact of deficiency in DNA non-homologous end-joining. DNA Repair.

[25] Riballo E., Kühne M., Rief N., Doherty A., Smith G.C., Recio M.a.-J., Reis C., Dahm K., Fricke A., Krempler A. (2004). A pathway of double-strand break rejoining dependent upon atm, artemis, and proteins locating to γ-h2ax foci. Mol. Cell.

[26] Sonoda E., Hochegger H., Saberi A., Taniguchi Y., Takeda S. (2006). Differential usage of non-homologous end-joining and homologous recombination in double strand break repair. DNA Repair.

[27] Beucher A., Birraux J., Tchouandong L., Barton O., Shibata A., Conrad S., Goodarzi A.A., Krempler A., Jeggo P.A., Löbrich M. (2009). Atm and artemis promote homologous recombination of radiation-induced DNA double-strand breaks in g2. EMBO J..

[28] Abbotts R., Wilson III D.M. (2017). Coordination of DNA single strand break repair. Free Radic. Biol. Med..

[29] Khoronenkova S.V., Dianov G.L. (2015). Atm prevents dsb formation by coordinating ssb repair and cell cycle progression. Proc. Natl. Acad. Sci. USA.

[30] Fortini P., Pascucci B., Parlanti E., D’errico M., Simonelli V., Dogliotti E. (2003). The base excision repair: Mechanisms and its relevance for cancer susceptibility. Biochimie.

[31] Czornak K., Chughtai S., Chrzanowska K.H. (2008). Mystery of DNA repair: The role of the mrn complex and atm kinase in DNA damage repair. J. Appl. Genet..

[32] Cimprich K.A., Cortez D. (2008). Atr: An essential regulator of genome integrity. Nat. Rev. Mol. Cell Biol..

[33] Shiloh Y. (2003). Atm and related protein kinases: Safeguarding genome integrity. Nat. Rev. Cancer.

[34] Kastan M.B., Onyekwere O., Sidransky D., Vogelstein B., Craig R.W. (1991). Participation of p53 protein in the cellular response to DNA damage. Cancer Res..

[35] Goto H., Izawa I., Li P., Inagaki M. (2012). Novel regulation of checkpoint kinase 1: Is checkpoint kinase 1 a good candidate for anti-cancer therapy?. Cancer Sci..

[36] Dasika G.K., Lin S.-C.J., Zhao S., Sung P., Tomkinson A., Lee E.Y.P. (1999). DNA damage-induced cell cycle checkpoints and DNA strand break repair in development and tumorigenesis. Oncogene.

[37] Paull T.T., Rogakou E.P., Yamazaki V., Kirchgessner C.U., Gellert M., Bonner W.M. (2000). A critical role for histone h2ax in recruitment of repair factors to nuclear foci after DNA damage. Curr. Biol..

[38] Rappold I., Iwabuchi K., Date T., Chen J. (2001). Tumor suppressor p53 binding protein 1 (53bp1) is involved in DNA damage–signaling pathways. J. Cell Biol..

[39] Paull T.T. (2015). Mechanisms of atm activation. Annu. Rev. Biochem..

[40] Burma S., Chen B.P., Murphy M., Kurimasa A., Chen D.J. (2001). Atm phosphorylates histone h2ax in response to DNA double-strand breaks. J. Biol. Chem..

[41] Ward I.M., Chen J. (2001). Histone h2ax is phosphorylated in an atr-dependent manner in response to replicational stress. J. Biol. Chem..

[42] Ward I.M., Minn K., Chen J. (2004). Uv-induced ataxia-telangiectasia-mutated and rad3-related (atr) activation requires replication stress. J. Biol. Chem..

[43] Bensimon A., Schmidt A., Ziv Y., Elkon R., Wang S.-Y., Chen D.J., Aebersold R., Shiloh Y. (2010). Atm-dependent and-independent dynamics of the nuclear phosphoproteome after DNA damage. Sci. Signal..

[44] Bakkenist C.J., Kastan M.B. (2003). DNA damage activates atm through intermolecular autophosphorylation and dimer dissociation. Nature.

[45] Guo Z., Kozlov S., Lavin M.F., Person M.D., Paull T.T. (2010). Atm activation by oxidative stress. Science.

[46] Canman C.E., Lim D.-S., Cimprich K.A., Taya Y., Tamai K., Sakaguchi K., Appella E., Kastan M.B., Siliciano J.D. (1998). Activation of the atm kinase by ionizing radiation and phosphorylation of p53. Science.

[47] Bodgi L., Granzotto A., Devic C., Vogin G., Lesne A., Bottollier-Depois J.-F., Victor J.-M., Maalouf M., Fares G., Foray N. (2013). A single formula to describe radiation-induced protein relocalization: Towards a mathematical definition of individual radiosensitivity. J. Theor. Biol..

[48] Ouenzar F., Hendzel M.J., Weinfeld M. (2016). Shuttling towards a predictive assay for radiotherapy. Transl. Cancer Res..

[49] Ferlazzo M.L., Sonzogni L., Granzotto A., Bodgi L., Lartin O., Devic C., Vogin G., Pereira S., Foray N. (2014). Mutations of the huntington’s disease protein impact on the atm-dependent signaling and repair pathways of the radiation-induced DNA double-strand breaks: Corrective effect of statins and bisphosphonates. Mol. Neurobiol..

[50] Pereira S., Bodgi L., Duclos M., Canet A., Ferlazzo M.L., Devic C., Granzotto A., Deneuve S., Vogin G., Foray N. (2018). Fast and binary assay for predicting radiosensitivity based on the theory of atm nucleo-shuttling: Development, validation, and performance. Int. J. Radiat. Oncol. Biol. Phys..

[51] Ferlazzo M.L., Bach-Tobdji M.K.E., Djerad A., Sonzogni L., Devic C., Granzotto A., Bodgi L., Bachelet J.-T., Djefal-Kerrar A., Hennequin C. (2018). Radiobiological characterization of tuberous sclerosis: A delay in the nucleo-shuttling of atm may be responsible for radiosensitivity. Mol. Neurobiol..

[52] Surova O., Zhivotovsky B. (2013). Various modes of cell death induced by DNA damage. Oncogene.

[53] Lombard D.B., Chua K.F., Mostoslavsky R., Franco S., Gostissa M., Alt F.W. (2005). DNA repair, genome stability, and aging. Cell.

[54] Hasty P., Campisi J., Hoeijmakers J., Van Steeg H., Vijg J. (2003). Aging and genome maintenance: Lessons from the mouse?. Science.

[55] Tchkonia T., Zhu Y., Van Deursen J., Campisi J., Kirkland J.L. (2013). Cellular senescence and the senescent secretory phenotype: Therapeutic opportunities. J. Clin. Investig..

[56] Baskar R., Lee K.A., Yeo R., Yeoh K.-W. (2012). Cancer and radiation therapy: Current advances and future directions. Int. J. Med Sci..

[57] Eriksson D., Stigbrand T. (2010). Radiation-induced cell death mechanisms. Tumor Biol..

[58] Hollstein M., Sidransky D., Vogelstein B., Harris C.C. (1991). P53 mutations in human cancers. Science.

[59] Soussi T., Béroud C. (2001). Assessing tp53 status in human tumours to evaluate clinical outcome. Nat. Rev. Cancer.

[60] Soussi T., Lozano G. (2005). P53 mutation heterogeneity in cancer. Biochem. Biophys. Res. Commun..

[61] Helton E.S., Chen X. (2007). P53 modulation of the DNA damage response. J. Cell. Biochem..

[62] Wang Z., Li B. (2010). Mdm2 links genotoxic stress and metabolism to p53. Protein Cell.

[63] Fu W., Ma Q., Chen L., Li P., Zhang M., Ramamoorthy S., Nawaz Z., Shimojima T., Wang H., Yang Y. (2009). Mdm2 acts downstream of p53 as an e3 ligase to promote foxo ubiquitination and degradation. J. Biol. Chem..

[64] Barak Y., Gottlieb E., Juven-Gershon T., Oren M. (1994). Regulation of mdm2 expression by p53: Alternative promoters produce transcripts with nonidentical translation potential. Genes Dev..

[65] Iwakuma T., Lozano G. (2003). Mdm2, an introduction. Mol. Cancer Res..

[66] Igney F.H., Krammer P.H. (2002). Death and anti-death: Tumour resistance to apoptosis. Nat. Rev. Cancer.

[67] Gewirtz D.A., Holt S.E., Elmore L.W. (2008). Accelerated senescence: An emerging role in tumor cell response to chemotherapy and radiation. Biochem. Pharmacol..

[68] Rodier F., Coppé J.-P., Patil C.K., Hoeijmakers W.A., Muñoz D.P., Raza S.R., Freund A., Campeau E., Davalos A.R., Campisi J. (2009). Persistent DNA damage signalling triggers senescence-associated inflammatory cytokine secretion. Nat. Cell Biol..

[69] Batalni J.P., Belloir C., Mazabraud A., Pilleron J.P., Cartigny A., Jaulerry C., Ghossein N.A. (1988). Desmoid tumors in adults: The role of radiotherapy in their management. Am. J. Surg..

[70] Cox J.D., Kline R.W. (1983). Do prostatic biopsies 12 months or more after external irradiation for adenocarcinoma, stage iii, predict long-term survival?. Int. J. Radiat. Oncol. Biol. Phys..

[71] Shay J.W., Roninson I.B. (2004). Hallmarks of senescence in carcinogenesis and cancer therapy. Oncogene.

[72] Suzuki K., Mori I., Nakayama Y., Miyakoda M., Kodama S., Watanabe M. (2001). Radiation-induced senescence-like growth arrest requires tp53 function but not telomere shortening. Radiat. Res..

[73] Ninomiya Y., Cui X., Yasuda T., Wang B., Yu D., Sekine-Suzuki E., Nenoi M. (2014). Arsenite induces premature senescence via p53/p21 pathway as a result of DNA damage in human malignant glioblastoma cells. BMB Rep..

[74] Pawlowska E., Szczepanska J., Szatkowska M., Blasiak J. (2018). An interplay between senescence, apoptosis and autophagy in glioblastoma multiforme—Role in pathogenesis and therapeutic perspective. Int. J. Mol. Sci..

[75] Hock A.K., Vousden K.H. (2012). Tumor suppression by p53: Fall of the triumvirate?. Cell.

[76] Kim H., Lee J.M., Lee G., Bhin J., Oh S.K., Kim K., Pyo K.E., Lee J.S., Yim H.Y., Kim K.I. (2011). DNA damage-induced rorα is crucial for p53 stabilization and increased apoptosis. Mol. Cell.

[77] Baliga B.C., Colussi P.A., Read S.H., Dias M.M., Jans D.A., Kumar S. (2003). Role of prodomain in importin-mediated nuclear localization and activation of caspase-2. J. Biol. Chem..

[78] Tinel A., Tschopp J. (2004). The piddosome, a protein complex implicated in activation of caspase-2 in response to genotoxic stress. Science.

[79] Schmitt C.A. (2003). Senescence, apoptosis and therapy—Cutting the lifelines of cancer. Nat. Rev. Cancer.

[80] Galluzzi L., Maiuri M., Vitale I., Zischka H., Castedo M., Zitvogel L., Kroemer G. (2007). Cell Death Modalities: Classification and Pathophysiological Implications. Cell Death Differ..

[81] Eriksson D., Joniani H.M., Sheikholvaezin A., Löfroth P.-O., Johansson L., Åhlström K.R., Stigbrand T. (2003). Combined low dose radio-and radioimmunotherapy of experimental hela hep 2 tumours. Eur. J. Nucl. Med. Mol. Imaging.

[82] Eriksson D., Löfroth P.-O., Johansson L., Riklund K.Å., Stigbrand T. (2007). Cell cycle disturbances and mitotic catastrophes in hela hep2 cells following 2.5 to 10 gy of ionizing radiation. Clin. Cancer Res..

[83] Castedo M., Kroemer G. (2004). Mitotic catastrophe: A special case of apoptosis. J. Soc. Biol..

[84] Erenpreisa J., Kalejs M., Ianzini F., Kosmacek E.A., Mackey M., Emzinsh D., Cragg M.S., Ivanov A., Illidge T.M. (2005). Segregation of genomes in polyploid tumour cells following mitotic catastrophe. Cell Biol. Int..

[85] Roninson I.B., Broude E.V., Chang B.-D. (2001). If not apoptosis, then what? Treatment-induced senescence and mitotic catastrophe in tumor cells. Drug Resist. Updates.

[86] Ianzini F., Bertoldo A., Kosmacek E.A., Phillips S.L., Mackey M.A. (2006). Lack of p53 function promotes radiation-induced mitotic catastrophe in mouse embryonic fibroblast cells. Cancer Cell Int..

[87] Bourke E., Dodson H., Merdes A., Cuffe L., Zachos G., Walker M., Gillespie D., Morrison C.G. (2007). DNA damage induces chk1-dependent centrosome amplification. EMBO Rep..

[88] Dodson H., Wheatley S.P., Morrison C.G. (2007). Involvement of centrosome amplification in radiation-induced mitotic catastrophe. Cell Cycle.

[89] Kawamura K., Fujikawa-Yamamoto K., Ozaki M., Iwabuchi K., Nakashima H., Domiki C., Morita N., Inoue M., Tokunaga K., Shiba N. (2004). Centrosome hyperamplification and chromosomal damage after exposure to radiation. Oncology.

[90] Kawamura K., Morita N., Domiki C., Fujikawa-Yamamoto K., Hashimoto M., Iwabuchi K., Suzuki K. (2006). Induction of centrosome amplification in p53 sirna-treated human fibroblast cells by radiation exposure. Cancer Sci..

[91] Hanashiro K., Kanai M., Geng Y., Sicinski P., Fukasawa K. (2008). Roles of cyclins a and e in induction of centrosome amplification in p53-compromised cells. Oncogene.

[92] Vakifahmetoglu H., Olsson M., Zhivotovsky B. (2008). Death through a tragedy: Mitotic catastrophe. Cell Death Differ..

[93] Kroemer G., Galluzzi L., Vandenabeele P., Abrams J., Alnemri E.S., Baehrecke E., Blagosklonny M., El-Deiry W., Golstein P., Green D. (2009). Classification of cell death: Recommendations of the nomenclature committee on cell death 2009. Cell Death Differ..

[94] Brandsma D., Stalpers L., Taal W., Sminia P., van den Bent M.J. (2008). Clinical features, mechanisms, and management of pseudoprogression in malignant gliomas. Lancet Oncol..

[95] Krysko O., Aaes T.L., Bachert C., Vandenabeele P., Krysko D. (2013). Many faces of damps in cancer therapy. Cell Death Dis..

[96] Proskuryakov S.Y., Konoplyannikov A.G., Gabai V.L. (2003). Necrosis: A specific form of programmed cell death?. Exp. Cell Res..

[97] Cohen–Jonathan E., Bernhard E.J., McKenna W.G. (1999). How does radiation kill cells?. Curr. Opin. Chem. Biol..

[98] Gault C.R., Obeid L.M., Hannun Y.A. (2010). An overview of sphingolipid metabolism: From synthesis to breakdown. Sphingolipids as Signaling and Regulatory Molecules.

[99] Abou Daher A., El Jalkh T., Eid A., Fornoni A., Marples B., Zeidan Y. (2017). Translational aspects of sphingolipid metabolism in renal disorders. Int. J. Mol. Sci..

[100] Zeidan Y.H., Hannun Y.A. (2007). Translational aspects of sphingolipid metabolism. Trends Mol. Med..

[101] Hannun Y.A., Obeid L.M. (2008). Principles of bioactive lipid signalling: Lessons from sphingolipids. Nat. Rev. Mol. Cell Biol..

[102] Fröhlich F., Petit C., Kory N., Christiano R., Hannibal-Bach H.-K., Graham M., Liu X., Ejsing C.S., Farese R.V., Walther T.C. (2015). The garp complex is required for cellular sphingolipid homeostasis. Elife.

[103] te Vruchte D., Lloyd-Evans E., Veldman R.J., Neville D.C., Dwek R.A., Platt F.M., van Blitterswijk W.J., Sillence D.J. (2004). Accumulation of glycosphingolipids in niemann-pick c disease disrupts endosomal transport. J. Biol. Chem..

[104] Höglinger D., Haberkant P., Aguilera-Romero A., Riezman H., Porter F.D., Platt F.M., Galione A., Schultz C. (2015). Intracellular sphingosine releases calcium from lysosomes. Elife.

[105] Schuchman E. (2007). The pathogenesis and treatment of acid sphingomyelinase-deficient niemann–pick disease. J. Inherit. Metab. Dis..

[106] Merscher S., Fornoni A. (2014). Podocyte pathology and nephropathy–sphingolipids in glomerular diseases. Front. Endocrinol..

[107] Boath A., Graf C., Lidome E., Ullrich T., Nussbaumer P., Bornancin F. (2008). Regulation and traffic of ceramide 1-phosphate produced by ceramide kinase comparative analysis to glucosylceramide and sphingomyelin. J. Biol. Chem..

[108] Ogawa C., Kihara A., Gokoh M., Igarashi Y. (2003). Identification and characterization of a novel human sphingosine-1-phosphate phosphohydrolase, hspp2. J. Biol. Chem..

[109] Mandala S.M., Thornton R., Galve-Roperh I., Poulton S., Peterson C., Olivera A., Bergstrom J., Kurtz M.B., Spiegel S. (2000). Molecular cloning and characterization of a lipid phosphohydrolase that degrades sphingosine-1-phosphate and induces cell death. Proc. Natl. Acad. Sci. USA.

[110] Pyne S., Long J., Ktistakis N., Pyne N. (2005). Lipid phosphate phosphatases and lipid phosphate signalling. Biochem. Soc. Trans..

[111] Ikeda M., Kihara A., Igarashi Y. (2004). Sphingosine-1-phosphate lyase spl is an endoplasmic reticulum-resident, integral membrane protein with the pyridoxal 5′-phosphate binding domain exposed to the cytosol. Biochem. Biophys. Res. Commun..

[112] Albi E. (2011). Role of intranuclear lipids in health and disease. Clin. Lipidol..

[113] Ledeen R.W., Wu G. (2008). Thematic review series: Sphingolipids. Nuclear sphingolipids: Metabolism and signaling. J. Lipid Res..

[114] Bernardini I., Bartoccini E., Viola Magni M. (2017). Nuclear lipids and cell fate. Dyn. Cell Biol..

[115] Divecha N., Banfic H., Irvine R.F. (1993). Inositides and the nucleus and inositides in the nucleus. Cell.

[116] Cocco L., Martelli A.M., Gilmour R.S., Rhee S.G., Manzoli F.A. (2001). Nuclear phospholipase c and signaling. Biochim. Biophys. Acta.

[117] Maraldi N., Cocco L., Capitani S., Mazzotti G., Barnabei O., Manzoli F. (1994). Lipid-dependent nuclear signalling: Morphological and functional features. Adv. Enzym. Regul..

[118] Kleuser B., Maceyka M., Milstien S., Spiegel S. (2001). Stimulation of nuclear sphingosine kinase activity by platelet-derived growth factor. FEBS Lett..

[119] Ledeen R.W., Wu G. (2006). Sphingolipids of the nucleus and their role in nuclear signaling. Biochim. Biophys. Acta (BBA) Mol. Cell Biol. Lipids.

[120] Neitcheva T., Peeva D. (1995). Phospholipid composition, phospholipase a2 and sphingomyelinase activities in rat liver nuclear membrane and matrix. Int. J. Biochem. Cell Biol..

[121] Pliss A., Kuzmin A.N., Kachynski A.V., Prasad P.N. (2010). Nonlinear optical imaging and raman microspectrometry of the cell nucleus throughout the cell cycle. Biophys. J..

[122] Albi E., Mersel M., Leray C., Tomassoni M., Viola-Magni M. (1994). Rat liver chromatin phospholipids. Lipids.

[123] Cave C.F., Gahan P. (1970). A cytochemical and autoradiographic investigation of nucleolar phospholipids. Caryologia.

[124] Fu P., Ebenezer D.L., Ha A.W., Suryadevara V., Harijith A., Natarajan V. (2018). Nuclear lipid mediators: Role of nuclear sphingolipids and sphingosine-1-phosphate signaling in epigenetic regulation of inflammation and gene expression. J. Cell. Biochem..

[125] Tomassoni M.-L., Amori D., Magni M.V. (1999). Changes of nuclear membrane lipid composition affect rna nucleocytoplasmic transport. Biochem. Biophys. Res. Commun..

[126] Albi E., Tomassoni M.L., Viola-Magni M. (1997). Effect of lipid composition on rat liver nuclear membrane fluidity. Cell Biochem. Funct. Cell. Biochem. Modul. Act. Agents Dis..

[127] Xie X., Wu G., Lu Z.H., Ledeen R.W. (2002). Potentiation of a sodium–calcium exchanger in the nuclear envelope by nuclear gm1 ganglioside. J. Neurochem..

[128] Albi E., Magni M.V. (2003). Chromatin-associated sphingomyelin: Metabolism in relation to cell function. Cell Biochem. Funct. Cell. Biochem. Modul. Act. Agents Dis..

[129] Lucki N.C., Sewer M.B. (2012). Nuclear sphingolipid metabolism. Annu. Rev. Physiol..

[130] Alessenko A., Chatterjee S. (1995). Neutral sphingomyelinase: Localization in rat liver nuclei and involvement in regeneration/proliferation. Mol. Cell. Biochem..

[131] Wu G., Lu Z.H., Ledeen R.W. (1995). Gm1 ganglioside in the nuclear membrane modulates nuclear calcium homeostasis during neurite outgrowth. J. Neurochem..

[132] Micheli M., Albi E., Leray C., Magni M.V. (1998). Nuclear sphingomyelin protects rna from rnase action. FEBS Lett..

[133] Tsugane K., Tamiya-Koizumi K., Nagino M., Nimura Y., Yoshida S. (1999). A possible role of nuclear ceramide and sphingosine in hepatocyte apoptosis in rat liver. J. Hepatol..

[134] Rossi G., Magni M.V., Albi E. (2007). Sphingomyelin-cholesterol and double stranded rna relationship in the intranuclear complex. Arch. Biochem. Biophys..

[135] Albi E., Cataldi S., Rossi G., Magni M.V., Toller M., Casani S., Perrella G. (2008). The nuclear ceramide/diacylglycerol balance depends on the physiological state of thyroid cells and changes during uv-c radiation-induced apoptosis. Arch. Biochem. Biophys..

[136] Hait N.C., Allegood J., Maceyka M., Strub G.M., Harikumar K.B., Singh S.K., Luo C., Marmorstein R., Kordula T., Milstien S. (2009). Regulation of histone acetylation in the nucleus by sphingosine-1-phosphate. Science.

[137] Gupta S., Maurya M.R., Merrill A.H., Glass C.K., Subramaniam S. (2011). Integration of lipidomics and transcriptomics data towards a systems biology model of sphingolipid metabolism. BMC Syst. Biol..

[138] Albi E., Viola Magni M. (2006). Sphingomyelin: A small-big molecule in the nucleus. Recent Res. Dev. Biophys. Biochem..

[139] Exton J. (1990). Signaling through phosphatidylcholine breakdown. J. Biol. Chem..

[140] Reszka A.A., Halasy-Nagy J., Rodan G.A. (2001). Nitrogen-bisphosphonates block retinoblastoma phosphorylation and cell growth by inhibiting the cholesterol biosynthetic pathway in a keratinocyte model for esophageal irritation. Mol. Pharmacol..

[141] Novello F., Muchmore J., Bonora B., Capitani S., Manzoli F. (1975). Effect of phospholipids on the activity of DNA polymerase i from e. Coli. Ital. J. Biochem..

[142] Martelli A.M., Follo M.Y., Evangelisti C., Fala F., Fiume R., Billi A.M., Cocco L. (2005). Nuclear inositol lipid metabolism: More than just second messenger generation?. J. Cell. Biochem..

[143] Scassellati C., Albi E., Cmarko D., Tiberi C., Cmarkova J., Bouchet-Marquis C., Verschure P.J., Van Driel R., Magni M.V., Fakan S. (2010). Intranuclear sphingomyelin is associated with transcriptionally active chromatin and plays a role in nuclear integrity. Biol. Cell.

[144] Albi E., Lazzarini R., Magni M.V. (2003). Reverse sphingomyelin-synthase in rat liver chromatin. FEBS Lett..

[145] Albi E., Magni M.V. (1999). Sphingomyelin synthase in rat liver nuclear membrane and chromatin. FEBS Lett..

[146] Albi E., Magni M.V. (1997). Chromatin neutral sphingomyelinase and its role in hepatic regeneration. Biochem. Biophys. Res. Commun..

[147] Venkataraman K., Riebeling C., Bodennec J., Riezman H., Allegood J.C., Sullards M.C., Merrill A.H., Futerman A.H. (2002). Upstream of growth and differentiation factor 1 (uog1), a mammalian homolog of the yeast longevity assurance gene 1 (lag1), regulatesn-stearoyl-sphinganine (c18-(dihydro) ceramide) synthesis in a fumonisin b1-independent manner in mammalian cells. J. Biol. Chem..

[148] Riebeling C., Allegood J.C., Wang E., Merrill A.H., Futerman A.H. (2003). Two mammalian longevity assurance gene (lag1) family members, trh1 and trh4, regulate dihydroceramide synthesis using different fatty acyl-coa donors. J. Biol. Chem..

[149] Mizutani Y., Kihara A., Igarashi Y. (2005). Mammalian lass6 and its related family members regulate synthesis of specific ceramides. Biochem. J..

[150] Min J., Mesika A., Sivaguru M., Van Veldhoven P.P., Alexander H., Futerman A.H., Alexander S. (2007). (dihydro) ceramide synthase 1–regulated sensitivity to cisplatin is associated with the activation of p38 mitogen-activated protein kinase and is abrogated by sphingosine kinase 1. Mol. Cancer Res..

[151] Shiraishi T., Imai S., Uda Y. (2003). The presence of ceramidase activity in liver nuclear membrane. Biol. Pharm. Bull..

[152] Watanabe M., Kitano T., Kondo T., Yabu T., Taguchi Y., Tashima M., Umehara H., Domae N., Uchiyama T., Okazaki T. (2004). Increase of nuclear ceramide through caspase-3-dependent regulation of the “sphingomyelin cycle” in fas-induced apoptosis. Cancer Res..

[153] Albi E., Cataldi S., Bartoccini E., Magni M.V., Marini F., Mazzoni F., Rainaldi G., Evangelista M., Garcia-Gil M. (2006). Nuclear sphingomyelin pathway in serum deprivation-induced apoptosis of embryonic hippocampal cells. J. Cell. Physiol..

[154] Chocian G., Chabowski A., Żendzian-Piotrowska M., Harasim E., Łukaszuk B., Górski J. (2010). High fat diet induces ceramide and sphingomyelin formation in rat’s liver nuclei. Mol. Cell. Biochem..

[155] Schroeder F., Petrescu A.D., Huang H., Atshaves B.P., McIntosh A.L., Martin G.G., Hostetler H.A., Vespa A., Landrock D., Landrock K.K. (2008). Role of fatty acid binding proteins and long chain fatty acids in modulating nuclear receptors and gene transcription. Lipids.

[156] Yamaji T., Kumagai K., Tomishige N., Hanada K. (2008). Two sphingolipid transfer proteins, cert and fapp2: Their roles in sphingolipid metabolism. Iubmb Life.

[157] Sugiura M., Kono K., Liu H., Shimizugawa T., Minekura H., Spiegel S., Kohama T. (2002). Ceramide kinase, a novel lipid kinase molecular cloning and functional characterization. J. Biol. Chem..

[158] Simanshu D.K., Kamlekar R.K., Wijesinghe D.S., Zou X., Zhai X., Mishra S.K., Molotkovsky J.G., Malinina L., Hinchcliffe E.H., Chalfant C.E. (2013). Non-vesicular trafficking by a ceramide-1-phosphate transfer protein regulates eicosanoids. Nature.

[159] Rovina P., Schanzer A., Graf C., Mechtcheriakova D., Jaritz M., Bornancin F. (2009). Subcellular localization of ceramide kinase and ceramide kinase-like protein requires interplay of their pleckstrin homology domain-containing n-terminal regions together with c-terminal domains. Biochim. Biophys. Acta (BBA) Mol. Cell Biol. Lipids.

[160] Urs A.N., Dammer E., Kelly S., Wang E., Merrill A.H., Sewer M.B. (2007). Steroidogenic factor-1 is a sphingolipid binding protein. Mol. Cell. Endocrinol..

[161] Urs A.N., Dammer E., Sewer M.B. (2006). Sphingosine regulates the transcription of cyp17 by binding to steroidogenic factor-1. Endocrinology.

[162] Sewer M.B., Waterman M.R. (2003). Acth modulation of transcription factors responsible for steroid hydroxylase gene expression in the adrenal cortex. Microsc. Res. Tech..

[163] Spiegel S., Milstien S. (2007). Functions of the multifaceted family of sphingosine kinases and some close relatives. J. Biol. Chem..

[164] Maceyka M., Sankala H., Hait N.C., Le Stunff H., Liu H., Toman R., Collier C., Zhang M., Satin L.S., Merrill A.H. (2005). Sphk1 and sphk2, sphingosine kinase isoenzymes with opposing functions in sphingolipid metabolism. J. Biol. Chem..

[165] Alemany R., van Koppen C.J., Danneberg K., Ter Braak M., Zu Heringdorf D.M. (2007). Regulation and functional roles of sphingosine kinases. Naunyn-Schmiedeberg’s Arch. Pharmacol..

[166] Selvam S.P., De Palma R.M., Oaks J.J., Oleinik N., Peterson Y.K., Stahelin R.V., Skordalakes E., Ponnusamy S., Garrett-Mayer E., Smith C.D. (2015). Binding of the sphingolipid s1p to htert stabilizes telomerase at the nuclear periphery by allosterically mimicking protein phosphorylation. Sci. Signal..

[167] Stunff H.L., Milstien S., Spiegel S. (2004). Generation and metabolism of bioactive sphingosine-1-phosphate. J. Cell. Biochem..

[168] Wang C., Mao J., Redfield S., Mo Y., Lage J.M., Zhou X. (2014). Systemic distribution, subcellular localization and differential expression of sphingosine-1-phosphate receptors in benign and malignant human tissues. Exp. Mol. Pathol..

[169] Lépine S., Allegood J., Park M., Dent P., Milstien S., Spiegel S. (2011). Sphingosine-1-phosphate phosphohydrolase-1 regulates er stress-induced autophagy. Cell Death Differ..

[170] Schwiebs A., Thomas D., Kleuser B., Pfeilschifter J.M., Radeke H.H. (2017). Nuclear translocation of sgpp-1 and decrease of sgpl-1 activity contribute to sphingolipid rheostat regulation of inflammatory dendritic cells. Mediat. Inflamm..

[171] Ebenezer D., Fu P., Berdyshev E., Natarajan V. (2015). Nuclear s1p lyase regulates histone acetylation in pseudomonas aeruginosa-induced lung inflammation. FASEB J..

[172] Ebenezer D.L., Fu P., Mangio L.A., Berdyshev E., Schumacher F., Kleuser B., Van Veldhoven P.P., Natarajan V. (2019). Δ-2 hexadecenal generated from s1p by nuclear s1p lyase is a regulator of hdac1/2 activity and histone acetylation in lung epithelial cells. FASEB J..

[173] Reynolds C.P., Maurer B.J., Kolesnick R.N. (2004). Ceramide synthesis and metabolism as a target for cancer therapy. Cancer Lett..

[174] Gault C.R., Obeid L.M. (2011). Still benched on its way to the bedside: Sphingosine kinase 1 as an emerging target in cancer chemotherapy. Crit. Rev. Biochem. Mol. Biol..

[175] Dbaibo G.S., Pushkareva M.Y., Rachid R.A., Alter N., Smyth M.J., Obeid L.M., Hannun Y.A. (1998). P53-dependent ceramide response to genotoxic stress. J. Clin. Investig..

[176] Vit J.-P., Rosselli F. (2003). Role of the ceramide-signaling pathways in ionizing radiation-induced apoptosis. Oncogene.

[177] Sawada M., Nakashima S., Kiyono T., Nakagawa M., Yamada J., Yamakawa H., Banno Y., Shinoda J., Nishimura Y., Nozawa Y. (2001). P53 regulates ceramide formation by neutral sphingomyelinase through reactive oxygen species in human glioma cells. Oncogene.

[178] Corcoran C.A., He Q., Ponnusamy S., Ogretmen B., Huang Y., Sheikh M.S. (2008). Neutral sphingomyelinase-3 is a DNA damage and nongenotoxic stress-regulated gene that is deregulated in human malignancies. Mol. Cancer Res..

[179] Jaffrézou J.-P., Bruno A.P., Moisand A., Levade T., Laurent G. (2001). Activation of a nuclear sphingomyelinase in radiation-induced apoptosis. FASEB J..

[180] Ravid T., Tsaba A., Gee P., Rasooly R., Medina E.A., Goldkorn T. (2003). Ceramide accumulation precedes caspase-3 activation during apoptosis of a549 human lung adenocarcinoma cells. Am. J. Physiol. Lung Cell. Mol. Physiol..

[181] Dbaibo G.S., Pushkareva M.Y., Jayadev S., Schwarz J.K., Horowitz J.M., Obeid L.M., Hannun Y.A. (1995). Retinoblastoma gene product as a downstream target for a ceramide-dependent pathway of growth arrest. Proc. Natl. Acad. Sci. USA.

[182] Phillips D., Hunt J., Moneypenny C., Maclean K., McKenzie P., Harris L., Houghton J. (2007). Ceramide-induced g 2 arrest in rhabdomyosarcoma (rms) cells requires p21 cip1/waf1 induction and is prevented by mdm2 overexpression. Cell Death Differ..

[183] Xu R., Garcia-Barros M., Wen S., Li F., Lin C.-L., Hannun Y.A., Obeid L.M., Mao C. (2017). Tumor suppressor p53 links ceramide metabolism to DNA damage response through alkaline ceramidase 2. Cell Death Differ..

[184] Hoeferlin L.A., Fekry B., Ogretmen B., Krupenko S.A., Krupenko N.I. (2013). Folate stress induces apoptosis via p53-dependent de novo ceramide synthesis and up-regulation of ceramide synthase 6. J. Biol. Chem..

[185] Fekry B., Jeffries K.A., Esmaeilniakooshkghazi A., Szulc Z.M., Knagge K.J., Kirchner D.R., Horita D.A., Krupenko S.A., Krupenko N.I. (2018). C 16-ceramide is a natural regulatory ligand of p53 in cellular stress response. Nat. Commun..

[186] Taha T.A., Osta W., Kozhaya L., Bielawski J., Johnson K.R., Gillanders W.E., Dbaibo G.S., Hannun Y.A., Obeid L.M. (2004). Down-regulation of sphingosine kinase-1 by DNA damage dependence on proteases and p53. J. Biol. Chem..

[187] Sankala H.M., Hait N.C., Paugh S.W., Shida D., Lépine S., Elmore L.W., Dent P., Milstien S., Spiegel S. (2007). Involvement of sphingosine kinase 2 in p53-independent induction of p21 by the chemotherapeutic drug doxorubicin. Cancer Res..

[188] Johnson K.R., Johnson K.Y., Becker K.P., Bielawski J., Mao C., Obeid L.M. (2003). Role of human sphingosine-1-phosphate phosphatase 1 in the regulation of intra-and extracellular sphingosine-1-phosphate levels and cell viability. J. Biol. Chem..

[189] Oskouian B., Sooriyakumaran P., Borowsky A.D., Crans A., Dillard-Telm L., Tam Y.Y., Bandhuvula P., Saba J.D. (2006). Sphingosine-1-phosphate lyase potentiates apoptosis via p53-and p38-dependent pathways and is down-regulated in colon cancer. Proc. Natl. Acad. Sci. USA.

[190] Kumar A., Oskouian B., Fyrst H., Zhang M., Paris F., Saba J. (2011). S1p lyase regulates DNA damage responses through a novel sphingolipid feedback mechanism. Cell Death Dis..

[191] Ahmad A., Mitrofanova A., Bielawski J., Yang Y., Marples B., Fornoni A., Zeidan Y.H. (2016). Sphingomyelinase-like phosphodiesterase 3b mediates radiation-induced damage of renal podocytes. FASEB J..

[192] Fornoni A., Sageshima J., Wei C., Merscher-Gomez S., Aguillon-Prada R., Jauregui A.N., Li J., Mattiazzi A., Ciancio G., Chen L. (2011). Rituximab targets podocytes in recurrent focal segmental glomerulosclerosis. Sci. Transl. Med..

[193] Mitrofanova A., Mallela S., Ducasa G., Yoo T., Rosenfeld-Gur E., Zelnik I., Molina J., Santos J.V., Ge M., Sloan A. (2019). Smpdl3b modulates insulin receptor signaling in diabetic kidney disease. Nat. Commun..

[194] Mallela S.K., Mitrofanova A., Merscher S., Fornoni A. (2019). Regulation of the amount of ceramide-1-phosphate synthesized in differentiated human podocytes. Biochim. Biophys. Acta (BBA) Mol. Cell Biol. Lipids.

[195] Beckham T.H., Cheng J.C., Marrison S.T., Norris J.S., Liu X. (2013). Interdiction of sphingolipid metabolism to improve standard cancer therapies. Advances in Cancer Research.

[196] Savić R., Schuchman E.H. (2013). Use of acid sphingomyelinase for cancer therapy. Advances in Cancer Research.

[197] Albi E., Cataldi S., Ceccarini M.R., Conte C., Ferri I., Fettucciari K., Patria F.F., Beccari T., Codini M. (2019). Gentamicin targets acid sphingomyelinase in cancer: The case of the human gastric cancer nci-n87 cells. Int. J. Mol. Sci..

[198] Cao M., Ji C., Zhou Y., Huang W., Ni W., Tong X., Wei J.-F. (2018). Sphingosine kinase inhibitors: A patent review. Int. J. Mol. Med..

[199] Dubois N., Rio E., Ripoche N., Ferchaud-Roucher V., Gaugler M.-H., Campion L., Krempf M., Carrie C., Mahé M., Mirabel X. (2016). Plasma ceramide, a real-time predictive marker of pulmonary and hepatic metastases response to stereotactic body radiation therapy combined with irinotecan. Radiother. Oncol..

[200] Gao P., Smith C.D. (2011). Ablation of sphingosine kinase-2 inhibits tumor cell proliferation and migration. Mol. Cancer Res..

[201] Britten C.D., Garrett-Mayer E., Chin S.H., Shirai K., Ogretmen B., Bentz T.A., Brisendine A., Anderton K., Cusack S.L., Maines L.W. (2017). A phase i study of abc294640, a first-in-class sphingosine kinase-2 inhibitor, in patients with advanced solid tumors. Clin. Cancer Res..

